# Computational discovery of pathway-level genetic vulnerabilities in non-small-cell lung cancer

**DOI:** 10.1093/bioinformatics/btw010

**Published:** 2016-01-10

**Authors:** Jonathan H. Young, Michael Peyton, Hyun Seok Kim, Elizabeth McMillan, John D. Minna, Michael A. White, Edward M. Marcotte

**Affiliations:** ^1^Institute for Computational Engineering and Sciences, University of Texas at Austin, Austin, TX, USA,; ^2^Center for Systems and Synthetic Biology and Department of Molecular Biosciences, University of Texas at Austin, Austin, TX, USA,; ^3^Hamon Center for Therapeutic Oncology Research, University of Texas Southwestern Medical Center, Dallas, TX, USA,; ^4^Severance Biomedical Science Institute, Yonsei University College of Medicine, Seoul, Korea, and; ^5^Department of Cell Biology, University of Texas Southwestern Medical Center, Dallas, TX, USA

## Abstract

**Motivation:** Novel approaches are needed for discovery of targeted therapies for non-small-cell lung cancer (NSCLC) that are specific to certain patients. Whole genome RNAi screening of lung cancer cell lines provides an ideal source for determining candidate drug targets.

**Results:** Unsupervised learning algorithms uncovered patterns of differential vulnerability across lung cancer cell lines to loss of functionally related genes. Such genetic vulnerabilities represent candidate targets for therapy and are found to be involved in splicing, translation and protein folding. In particular, many NSCLC cell lines were especially sensitive to the loss of components of the LSm2-8 protein complex or the CCT/TRiC chaperonin. Different vulnerabilities were also found for different cell line subgroups. Furthermore, the predicted vulnerability of a single adenocarcinoma cell line to loss of the Wnt pathway was experimentally validated with screening of small-molecule Wnt inhibitors against an extensive cell line panel.

**Availability and implementation:** The clustering algorithm is implemented in Python and is freely available at https://bitbucket.org/youngjh/nsclc_paper.

**Contact:** marcotte@icmb.utexas.edu or jon.young@utexas.edu

**Supplementary information:** Supplementary data are available at *Bioinformatics* online.

## 1 Introduction

Non-small-cell lung cancer (NSCLC) remains a significant healthcare burden despite recent progress in drug discovery and development. Recent FDA-approved targeted therapies are only intended for appropriate subpopulations of patients. The drug Xalkori (crizotinib) is highly effective, but only for ∼4% of lung cancer patients (Shaw and Engelman, 2013). Similarly, Iressa (gefitinib) and other EGFR inhibitors target mutations found only in a portion of patients while the majority have the wild-type version (Laurie and Goss, 2013). Compared with cytotoxic chemotherapy, targeted therapy has the advantage of greater specificity. However, discovery and development of such agents requires the identification of druggable targets. Inhibitors of certain characteristic mutations, such as KRAS G12C and G12D, are still under extensive development for clinical use (Cox *et al.*, 2014; Hunter *et al.*, 2014). The heterogeneity of NSCLC is another barrier confronting drug discovery. A number of different subtypes exist, and identifying the appropriate patient subpopulation for therapy is crucial. Therefore, the problem becomes one of identifying druggable targets in NSCLC that will guide discovery of small-molecule compounds or antibodies against these targets, and also to identify the patient subpopulations to which the targets apply. We attempt to tackle the former issue through computational analysis of a high-throughput whole genome RNA interference (RNAi) screen against a panel of NSCLC cell lines.

When identifying drug targets, one approach is to identify genes whose knockdown selectively leads to death of cancer cells but not matched normal cells. Such genes represent genetic vulnerabilities and potential drug targets. Several studies have been able to use whole genome RNAi to identify genetic vulnerabilities for cancer drug target discovery. A screen against all genes in two lung cancer cell lines identified proteasome members as candidate targets and discovered that small-molecule proteasome inhibition synergized with radiotherapy in a mouse xenograft model (Cron *et al.*, 2013). Another study applied a whole genome shRNA screen on lung cancer cell lines to discover genes that were part of the Wnt pathway whose knockout potentiates EGFR inhibition (Casás-Selves *et al.*, 2012).

For drug discovery, it is desirable to investigate many cell lines. A major effort, termed Project Achilles, involved RNAi knockdown of more than 11 000 human genes using shRNA libraries in over 100 cancer cell lines. Ovarian cancer cell lines were found to be especially dependent upon ∼50 genes (Cheung *et al.*, 2011). A follow-up study sought to uncover essential cancer genes based on the hypothesis that certain genes that are not themselves oncogenes but show copy-number loss could be cancer vulnerabilities. A scoring scheme was developed to prioritize genes that were essential to cancer cell lines and also exhibited partial copy number loss (Nijhawan *et al.*, 2012). Recently, independent of Project Achilles, extensive high-throughput chemical and genetic screens were employed to explore new avenues of treating NSCLC. The study found molecular signatures of FLIP and COPI addiction and indolotriazine sensitivity that indicate genetic vulnerabilities present in patient populations (Kim *et al.*, 2013). The genetic screens leading to these results included siRNA screening of a number of lung cancer cell lines. This screening was ultimately conducted on a whole genome scale, which motivated this study.

Here, we propose a novel computational approach to prioritize candidate drug targets for NSCLC by subdividing cell lines into different groups and identifying genetic vulnerabilities targeted to each group. In particular, we aim to attain a binary partitioning of cell lines into either sensitive or resistant to targeting of a particular genetic vulnerability. We are interested only in genetic vulnerabilities that sensitize a subgroup of cell lines rather than all cell lines because due to the genetic heterogeneity of lung cancer, an effective universal treatment for all NSCLC types is not thought to exist. Applications of unsupervised learning algorithms were developed that identify biological processes and protein complexes to which NSCLC cell lines are differentially sensitive upon siRNA knockdown. The top-scoring results represent lung cancer genetic vulnerabilities and candidate therapy targets.

## 2 Methods

### 2.1 Experimental datasets and procedures

Our study centers on a cell line panel consisting of 12 patient-derived NSCLC cell lines and one immortalized normal epithelial cell line (Supplementary Table S1). Included among the cell lines are subtypes commonly observed in patients: adenocarcinoma, squamous-cell and large-cell carcinoma. As described previously (Kim *et al.*, 2013), a whole genome knockdown screen with Ambion and Dharmacon siRNA libraries in the 96-well plate format was conducted against the cell line panel. For each gene, either three siRNAs (Ambion) or four siRNAs (Dharmacon) were pooled, and cell line viability was measured using the CellTiter-Glo (Promega) assay. Raw data were row and column median normalized. Using siMacro (Singh *et al.*, 2013), a robust *Z* score was calculated from the screening data to reflect the viability of each cell line to knockdown of a single gene. A robust *Z* score is defined as

$$z=\frac{\text{Cell viability}-\text{median}}{\text{Median absolute deviation}}$$

and is less sensitive to outliers than a traditional *Z* score. Both the median and median absolute deviation were calculated over data grouped by experimental batch.

It was determined that robust *Z* scores less than −3.0 reflected non-viability. Scores were combined from both Ambion and Dharmacon libraries by taking the minimum of the scores. Thus, it was assumed that disagreement between the results of the two libraries were more likely to be due to false-negatives. The siRNA screen *Z* scores were further simplified by binarizing as follows. All robust *Z* scores less than −3.0 were set equal to 1; otherwise the score was set equal to 0. In essence, a binarized score of 1 represents a hit or sensitivity of a cell line to the corresponding gene knockdown.

A larger pool of NSCLC cell lines encompassing the cell line panel described above was screened with the tankyrase inhibitors IWR-1-endo (Calbiochem) and XAV 939 (Tocris) in an 8-point 4-fold dilution series (top dose = 100 µM) in 96-well plates. Cells were plated 24 h prior to the addition of drug, incubated for 4 days, and assayed using MTS (CellTiter 96 Aqueous One Solution Cell Proliferation Assay) according to the manufacturer’s instructions (Promega). Cell number per well was determined empirically and ranged from 500 to 4000 per well, inversely proportional to doubling times (typically 2000/well). Dose response curves were generated and IC50s calculated using in-house software, DIVISA. All cells were grown in RPMI-1640 (Sigma) supplemented with 5% FBS and incubated at 37°C in a humidified atmosphere with 5% CO_2_. Cell lines were authenticated using the Power-Plex 1.2 kit (Promega) and confirmed to match the DNA fingerprint library maintained by ATCC and the Minna/Gazdar laboratory and confirmed to be free of mycoplasma by e-Myco kit (Boca Scientific).

RNAi screens of cancer cell lines from Project Achilles (Cowley *et al.*, 2014) were utilized as an external comparison dataset for our study. Results from shRNA knockdown of 5711 genes on 19 NSCLC cell lines were extracted from Project Achilles v2.4.3. NaN values were imputed by replacement with row medians. No thresholding of the data was carried out so viability was assessed on a continuous spectrum. We followed the Project Achilles’ convention of identifying lower gene knockdown values with greater essentiality and higher values with reduced essentiality. A number of genes were associated with multiple knockdown values for each cell line; these data were kept as is.

### 2.2 Application of *k*-means clustering

The gene sets examined for genetic vulnerabilities were protein complexes chosen from CORUM (Ruepp *et al.*, 2010) and literature sources (Havugimana *et al.*, 2012; http://metazoa.med.utoronto.ca). The full RNAi data were represented as a m×n matrix *M* where *m* is the number of genes, *n* is the number of cell lines, and

$$M_{ij}=\{\begin{array}{ll} 0, & \text{if cell line }j\text{ survives knockdown of gene }i\\ 1, & \text{otherwise} \end{array}$$

Extracting the RNAi sensitivity profiles for genes in a protein complex yields a r×n submatrix *P* of *M* where *r* is the number of genes in the complex. Thus, every protein complex is represented as a matrix of ones and zeros.

For each protein complex, we measured the degree of bimodal response to gene knockdowns as follows. Denoting by *P* the matrix for a protein complex as above, we computed a vector *v* by calculating the column means of *P*: $P:v_{j}=\left(1/r\right)\sum_{i=1}^{r}P_{ij}$. Then *k*-means clustering with *k* = 2 was applied to *v*, and the difference between the resulting centroids was the score assigned to each protein complex. By calculating the column means, we normalized for the size of the complex. We considered two different implementations of the *k*-means clustering algorithm. First, we used the standard implementation found in the Python library *scikit-learn*, which runs the algorithm 10 times with different centroid seeds, choosing the result on the basis of the within-cluster sum-of-squares (Pedregosa *et al.*, 2011). The technique from *k*-means ++ was followed for centroid initialization (Arthur and Vassilvitskii, 2007). Second, an alternative implementation was *Ckmeans.1d.dp*, which employs dynamic programming to guarantee optimal solutions for the one-dimensional case (Wang and Song, 2011).

A permutation test to determine statistical significance was performed in the following manner. For each protein complex, the entire RNAi data for all genes were permuted, followed by repeating the *k*-means clustering on the same complex. The permutations were repeated 1000 times to generate a distribution of scores from the randomized data. Then the score from the actual complex was compared with the distribution to calculate a *P*-value. For every protein complex, this entire process of generating a distribution of scores from permuted data to compare against the complex’s actual score to yield a *P*-value was repeated. Finally, multiple hypothesis correction at 10% FDR was carried out using the *q*-value statistical package (Storey, 2002). One alternative to the permutation test in assessing statistical significance is Fisher’s exact test. Specifically, for each protein complex, a contingency table was tabulated according to the number of viable and non-viable gene knockdowns, and which cell line cluster (according to the 2-means clustering) those knockdowns fell within. The Bonferroni correction at 10% FDR was applied to the *P*-values from Fisher’s exact test.

A procedure to benchmark the performance of the 2-means clustering method was based on a leave-one-out strategy. For each protein complex, a single gene member was randomly withheld. The remaining gene members formed a training set, on which the 2-means clustering was calculated. The clustering resulted in assignments of cell lines to either a knockdown-sensitive or knockdown-resistant cluster. Using these assignments, we tested whether the average number of RNAi hits in the sensitive cell lines for the withheld gene was greater than that of the resistant lines. To assess for statistical significance in the training set, multiple hypothesis correction was performed using a permutation test as described above. A receiver operating characteristic (ROC) curve was plotted from the test set consisting of the withheld genes, given that the corresponding training samples were statistically significant. This benchmarking procedure was repeated multiple times and a mean ROC curve was generated by vertical averaging.

### 2.3 Biclustering

Independent of the 2-means clustering approach, a second method was employed to detect NSCLC genetic vulnerabilities without reliance on annotated gene sets. The entire RNAi knockdown dataset was represented as a matrix as described above, where each row is a gene knockdown and each column is a cell line. The Large Average Submatrix (LAS) biclustering algorithm was applied to this matrix to uncover biclusters in which the rows (genes) exhibit similar behavior across a set of columns (cell lines) (Shabalin *et al.*, 2009). In particular, the desired biclusters have the property of being large in average value relative to other submatrices of similar size and represent biological systems to which certain NSCLC cell lines are especially dependent. The genes corresponding to each bicluster were then used to query the Database for Annotation, Visualization and Integrated Discovery (DAVID) for functional enrichment (Huang *et al.*, 2009a, 2009b). We also searched each bicluster for enrichment of protein complexes by calculating the hypergeometric probability of obtaining at least the observed number of overlap between a complex and the bicluster genes. Statistical significance of complex enrichment was controlled at 5% FDR by the Benjamini–Hochberg procedure (Benjamini and Hochberg, 1995).

### 2.4 Alternative methods for determination of gene set sensitivity

We also considered alternative measures of gene set sensitivity. For every cell line within each gene set, the probability of observing the number of ‘hit’ genes (gene knockdowns producing non-viability) was computed according to a hypergeometric distribution. The resulting probabilities for each line were then multiplied together to obtain an overall score for the gene set. Statistical significance of the scores was found using a permutation test, as was done for the 2-means clustering.

## 3 Results

From a whole genome RNAi screen of NSCLC cell lines, we identified candidate drug targets in the form of genetic vulnerabilities specific to cell line subgroups. A couple of factors were considered when determining genetic vulnerabilities. First, given the heterogeneity of NSCLC, gene deletions that are almost universally toxic across the cell line panel would likely be toxic to other normal human cells as well. In addition, it is desirable to specifically target lung cancer cells but not normal cells, yet only one cell line in the panel was from a non-cancerous normal lineage. This suggests that in the interest of specificity, the number of cell lines constituting a genetic vulnerability should not be too large. On the other hand, a vulnerability consisting of a single cell line may be less likely to generalize to an appreciable number of patients. Therefore, the challenge stems from identifying groups of genes to which some, but not all, NSCLC cell lines are especially dependent. Ideally, these cell lines would represent a particular patient subpopulation. Another challenge was to avoid a combinatorially intractable problem of having to examine all possible combinations of cell lines against all possible combinations of genes. Novel applications of unsupervised learning algorithms were developed to overcome these challenges to prioritize potential NSCLC targets from RNAi sensitivities.

**Fig. 1.**
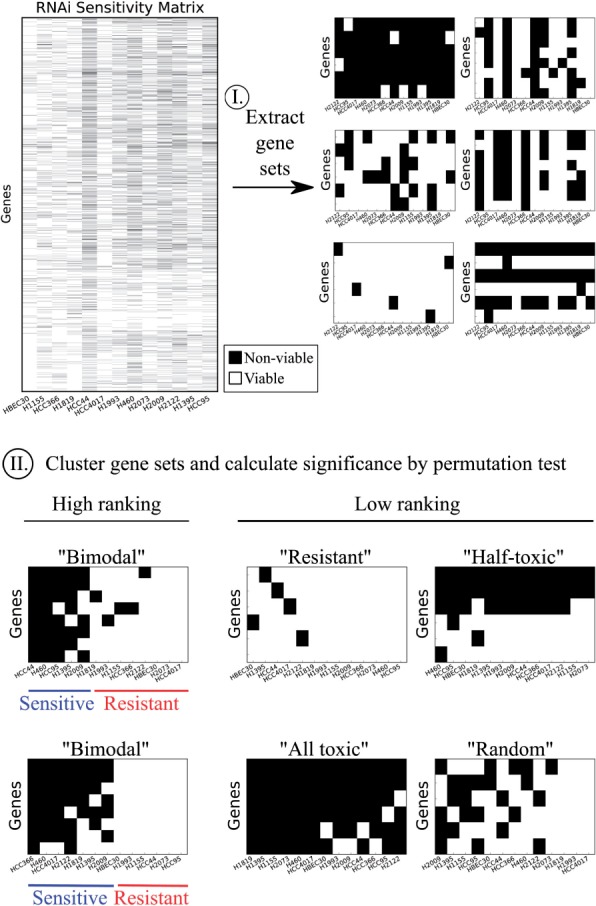
Gene sets with bimodal sensitivity represent NSCLC vulnerabilities. RNAi sensitivity profiles were extracted for selected gene sets (six examples shown). A ranking scheme was designed to prioritize gene sets whose knockdown leads to a bimodal response of cell lines

The general workflow of this study is outlined in Figure 1. From the whole genome RNAi screen on 12 NSCLC cell lines and one normal epithelial line, we extracted knockdown sensitivity profiles for selected gene sets. Each gene set was clustered, scored and ranked by statistical significance. The clustering score measures the degree to which the cell lines segregate into sensitive and resistant groups upon knockdown of genes in the set. Gene sets with a clear segregation of sensitive and resistant lines are termed bimodal. It is imperative that our scoring scheme prioritizes such bimodal sets over other patterns of RNAi sensitivity that would be less desirable as a candidate drug target. For example, gene sets that are all toxic or half-toxic are undesirable due to predicted toxicity beyond those in our cell line panel. In addition, gene sets that are largely resistant or having a random pattern of sensitivity clearly would not be desired as well.

### 3.1 Subgroup-specific NSCLC vulnerabilities are found among protein complexes

The selected gene sets we chose to examine were 2820 protein complexes. As calculated by the 2-means clustering approach, 35 had statistically significant scores at 10% FDR from a permutation test (Supplementary Fig. S1). Fisher’s exact test also determined 33 of those 35 complexes to be highly statistically significant (Supplementary Fig. S3c). We found that the standard *k*-means algorithm and the 1-D optimal *k*-means method, *Ckmeans.1d.dp*, yielded identical results although *Ckmeans.1d.dp* demonstrated marked runtime speedup. Finally, simulations of a synthetic dataset showed that the permutation test for statistical significance was not biased toward larger or smaller complexes (Supplementary Fig. S4).

**Fig. 2.**
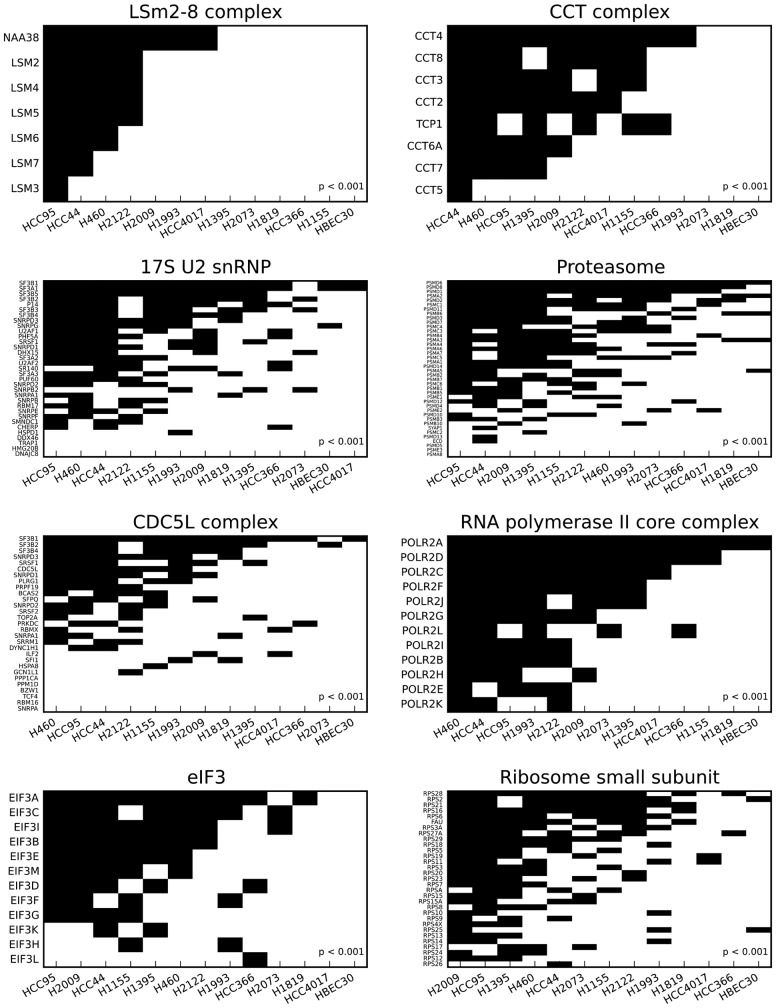
*k*-means clustering uncovers differential essentiality of NSCLC cell lines to protein complexes. The clustering partitions the cell line panel into two groups: sensitive and insensitive to loss of components of the complex. Shown are eight of 35 protein complexes that yielded statistically significant scores (10% FDR)

Overall, the 2-means clustering method found strong genetic vulnerabilities including components of splicing and translation (Fig. 2). The top-ranking gene sets are protein complexes that all exhibit the desired bimodal behavior, in which one particular group of cell lines is far more vulnerable to loss of components in the complex than the other cell lines. Notably, the HBEC30 normal cell line did not generally show sensitivity to knockdown of any of the top-ranking vulnerabilities.

RNA splicing is a major category of lung cancer vulnerabilities, as evidenced by the RNAi sensitivity patterns of the LSm2-8, 17S U2 snRNP and CDC5L complexes. Components of the translation machinery, represented by the eIF3 complex and ribosome small subunit, constitute another major class of vulnerabilities. Two notable candidate drug targets are the proteasome and the CCT/TRiC chaperonin complex, which gives one of the cleanest signals in terms of clustering the cell lines into sensitive and insensitive groups.

Moreover, different cell line subgroups exhibit different sensitivities. For example, HCC95, HCC44, H460 and H2122 are especially vulnerable to loss of the LSm2-8 complex, while a slightly broader cell line set is highly dependent upon the CCT complex. A few cell lines, particularly HCC95, are frequently sensitive to many of the genetic vulnerabilities. For some of the genetic vulnerabilities, the cell lines affected do not belong to a single histological subtype. Rather, they encompass at least one of the three main NSCLC subtypes of adenocarcinoma, squamous-cell and large-cell carcinoma.

In assessing the performance of our 2-means clustering by a leave-one-out strategy, only protein complexes above a certain size were considered. For complexes with at least five members, the 2-means clustering achieves a mean AUC of 0.62, with the average being computed over five iterations of withholding a random gene from each complex. When considering protein complexes containing at least eight members, a mean AUC of 0.66 is attained over eight iterations (Supplementary Fig. S2).

We applied our 2-means clustering method to data from Project Achilles on sensitivity of 19 NSCLC cell lines to shRNA knockdown of 5711 genes. Due to the lower coverage of genes compared to our whole genome knockdown dataset, the 2-means clustering is able to be applied to only a portion of many of the protein complexes. According to a permutation test, we were unable to find any statistically significant protein complexes at the same FDR 10% level previously used. The top scoring result is a ribosomal complex, followed by two proteasome complexes. A majority of the significant protein complexes found from our own RNAi dataset also maintain the general pattern of partitioning into sensitive and resistant cell lines in the Project Achilles experiment (Supplementary Fig. S1). Because no statistical significance was found, we did not carry out the benchmarking procedure as above.

### 3.2 Biclustering finds genetic vulnerabilities without reliance on annotated gene sets

LAS biclustering was employed as an independent and complementary approach to identifying candidate drug targets. The 2-means clustering approach relies on annotated gene sets, namely protein complexes, to address the challenge of selecting gene groups to interrogate for bimodal response to RNAi knockdown. On the other hand, biclustering offered an alternative strategy to tackle this challenge as it could find genetic vulnerabilities without regard to any prior annotation. The LAS algorithm found 22 statistically significant biclusters with Bonferroni-corrected *P*-values <10^−5^. Of the top 10 highest ranking biclusters, the first represents a nearly universally toxic set—all of the lung cancer cell lines are vulnerable to loss of almost any of the genes. The next best-ranking results are those which are toxic only to a single cell line. The lower-ranked statistically significant biclusters tend to represent vulnerabilities for a broader set of cell lines (Supplementary Table S2). Functional enrichment was not found for three of the top 10 results. In addition, searching each bicluster for enrichment of protein complexes yielded heavy enrichment for the spliceosome. For most of the biclusters, the functions of the enriched protein complexes match those found from the DAVID enrichment (Supplementary Table S3).

Many of the protein complexes from 2-means clustering also participate in biological processes found in LAS biclustering (Table 1). In particular, there appears to be frequent enrichment for translation and splicing, which are the functions of the ribosome small subunit, and the LSm2-8, CDC5L and 17S U2 snRNP complexes. No functional enrichment was found for the bicluster genes affecting HBEC30, which was also often resistant to knockdown of the protein complexes prioritized by 2-means clustering. Interestingly in 2-means clustering, HCC4017, HCC366, and H1819 were mostly among the groups of resistant cell lines although in biclustering, their genes were enriched in translation, splicing and proteasome components. Upon closer examination, the genes responsible for this enrichment are different from those comprising the translation, splicing and proteasome protein complexes. Apparently, biclustering complements the 2-means clustering in uncovering certain genetic vulnerabilities not found by the latter. We also note that the Wnt pathway, which is enriched in the 7^th^-ranked bicluster, was not discovered by the 2-means clustering as many of its genes either did not appear in our protein complex set or were only present individually in single complexes.

**Table 1.** Biclustering uncovers unique vulnerabilities of single NSCLC cell lines to biological processes

Bicluster rank	Size (genes × lines)	Lines affected	Enriched annotations
1	1591 × 12	All but HBEC30	Translation, splicing, kinetochores, mitosis
2	756 × 1	HBEC30	No enrichment
3	1060 × 1	HCC4017	Translation, splicing, nuclear lumen
4	1141 × 1	HCC366	Nuclear proteins and proteasome non-ATP subunits
5	1219 × 1	H1819	Translation, splicing
6	813 × 1	H1155	Nucleolar and cytoskeletal proteins
7	1154 × 1	H2073	Wnt pathway
8	859 × 2	H460, H2122	No enrichment
9	920 × 1	H1395	Translation
10	1254 × 1	H1993	No enrichment

The 10 highest scoring biclusters found from the LAS algorithm each represent genetic vulnerabilities for particular NSCLC cell lines. The genes in each bicluster were searched for functional enrichment.

### 3.3 Small-molecule screen confirms predicted cell line sensitivity

**Fig. 3.**
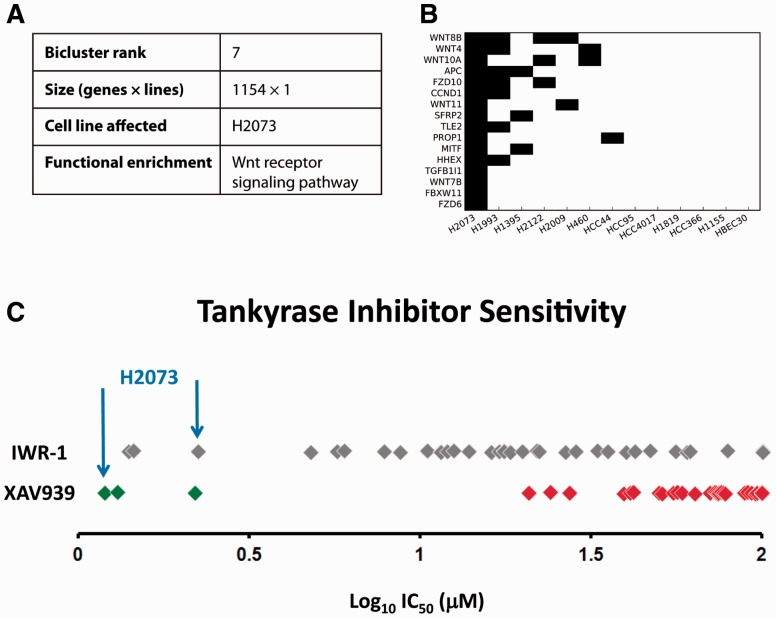
Biclustering finds strong vulnerability of H2073 to loss of Wnt signaling. (A) The seventh-ranked bicluster, containing 1154 genes, is enriched for the Wnt pathway. (B) The sensitivity profile of the gene set comprising the functional enrichment shows sensitivity of H2073 to knockdown of any of the genes in the set. (C) Screening of IWR-1 and XAV939 against an expanded panel of NSCLC cell lines (each denoted by a diamond) indeed shows that H2073 is markedly vulnerable to chemical inhibition of the Wnt pathway

One notable bicluster showed enrichment for the Wnt pathway (Fig. 3A and B). The H2073 adenocarcinoma cell was highly vulnerable to loss of Wnt pathway members. This suggests that small-molecule compounds targeting Wnt should reproduce the RNAi gene knockdown sensitivity pattern when tested on the cell line panel. Two Wnt inhibitors, IWR-1 and XAV939, were screened on a larger group of cell lines encompassing the panel, and the results confirmed the predicted sensitivity (Fig. 3C). The two compounds had a selective deleterious effect on H2073 while essentially sparing the other cell lines. Each cell line denoted by a diamond was colored according to a normal mixture model that predicted the number of groups. If there were two groups, green and red were used for sensitive and resistant, respectively. Otherwise, the diamonds were colored gray.

### 3.4 Alternative approaches to measuring complex sensitivity do not prioritize bimodality

We evaluated several other methods to measure complex sensitivity. These approaches depended on annotated gene sets, as opposed to biclustering, which has no such constraints. Cell line viability results from our whole genome screen are not approximately normally distributed (Supplementary Fig. S3a), which precludes the use of a simple *z*-test comparing the complex members’ scores to the background distribution. Even if the data were normally distributed (as the Project Achilles data is), this method would not distinguish bimodal from half-toxic complexes (Supplementary Fig. S3b), and in fact would prioritize universally toxic complexes.

We also considered using the hypergeometric distribution to assess the significance of multiple occurrences of sensitivity within a protein complex. From a permutation test, we found 544 statistically significant protein complexes at FDR 10% (Supplementary Fig. S3d). With the large number of protein complexes being statistically significant, we felt that this method was less discriminative than the 2-means clustering approach in prioritizing complexes.

## 4 Discussion

Collectively, the protein complexes we discovered to be NSCLC genetic vulnerabilities span various cellular processes including splicing, translation and protein folding. It is natural to ask how they fit in with currently established cancer therapies and whether known drugs could be repurposed for these complexes. Clearly, they contrast with hormonal therapy or the usual mitotic targets of cytotoxic chemotherapy. It turns out that some of the strongest genetic vulnerabilities are known targets of small-molecules.

Arsenic trioxide (As_2_O_3_) targets the TRiC/CCT complex (Pan *et al.*, 2010) and has been used to treat acute promyelocytic leukemia in patients who did not respond well to other types of chemotherapy (Shen *et al.*, 1997; Soignet *et al.*, 1998, 2001). As_2_O_3_ can perhaps be repurposed for NSCLC, particularly for patients whose tumors bear similarity to the sensitive cell line subgroups identified from the 2-means clustering analysis. Several studies have evaluated the effect of As_2_O_3_ in human lung primary fibroblasts and in the lung cancer cell lines A549 and H460 (Li *et al.*, 2009; Park and Kim, 2012). Collectively, they suggest that H460 is markedly more sensitive to As_2_O_3_ than lung fibroblasts, consistent with the CCT complex vulnerability we observed.

We also identified the proteasome as a candidate NSCLC drug target and recently, proteasome inhibitors have been investigated as anti-cancer agents. One such inhibitor is Velcade (bortezomib), which has been FDA-approved for multiple myeloma (Kisselev *et al.*, 2012). Bortezomib has shown to be effective in combination with other chemotherapy agents for NSCLC (Davies *et al.*, 2007) and has been evaluated in clinical trials for NSCLC as well (Besse *et al.*, 2012; Jones *et al.*, 2012; Piperdi *et al.*, 2012). This also suggests that newer and more specific proteasome inhibitors, such as Kyprolis (carfilzomib) could be efficacious for patients with NSCLC.

In addition, translation and splicing emerged as strong genetic vulnerabilities from the 2-means analysis. Previously, translation has been proposed as a potential target in cancer (Grzmil and Hemmings, 2012). Moreover, eIF3 is known to be overexpressed in lung cancers (Pincheira *et al.*, 2001), and ectopic expression of five eIF3 subunits has been shown to transform immortalized fibroblasts into malignant cells (Zhang *et al.*, 2007). Notably, in our study we found that knockdown of four of those five subunits strongly sensitizes six of the 12 NSCLC cell lines in our panel, while an immortalized epithelial line is comparatively unaffected (Fig. 2). The splicing apparatus has been suggested as a cancer target as well (Grosso *et al.*, 2008; van Alphen *et al.*, 2009). Of the splicing-associated protein complexes discovered from the 2-means analysis (Fig. 2), the SF3b component of U2 snRNP is known to be targeted by a number of small-molecule compounds. Both the pladienolides and meayamycin target SF3b, and the latter has been shown to be more deleterious in human lung cancer cells than normal lung fibroblasts (Albert *et al.*, 2009; Bonnal *et al.*, 2012).

Some of the NSCLC genetic vulnerabilities that were found by our computational analysis include protein complexes that may appear to be entirely essential. It is perhaps surprising that certain cell lines are largely resistant to knockdown of many of these genes. One explanation may simply be a result of the strict thresholding of the RNAi data to produce binary readings of cell line viability, which could be affected by the <100% sensitivity of the assay. Another explanation may be provided by essential gene ‘moonlighting’ and ‘flipping’ of protein complex essentiality between distantly related species (Ryan *et al.*, 2013). It was shown that certain protein complexes almost completely flip essentiality between *Saccharomyces cerevisiae* and *Schizosaccharomyces pombe*. A similar phenomenon may be occurring among our NSCLC cell line panel. Although the cell lines are not necessarily distantly related, they likely differ sufficiently due to different mutational compositions. Different yeast species flip protein complex essentiality as a result of adaptations to differing needs and environments, a phenomenon likely common to cancer cells as well. Moreover, particular NSCLC cell lines are largely resistant to loss of most, but not all, members of certain protein complexes. Those genes that are mostly essential in both sensitive and insensitive cell line subgroups could exhibit ‘moonlighting’ behavior by having multiple functions in both essential and nonessential processes.

The NSCLC genetic vulnerabilities uncovered by the computational analysis described here extends an earlier study (Kim *et al.*, 2013) in uncovering additional potential targets for therapy that were not previously reported. While our study shares the general aim of identifying genetic vulnerabilities, we exclusively focus on identification of biological systems, such as protein complexes, that certain lung cancers are especially dependent upon. Our results also present a complementary viewpoint to the Project Achilles effort in analyzing whole genome RNAi knockdown of cancer cells. One goal from Project Achilles was to discover genes that simultaneously had partial copy number loss and were essential to cancer cells (Nijhawan *et al.*, 2012). Interestingly, the results from that analysis found single gene vulnerabilities in splicing, translation and the proteasome as well. One such vulnerability was LSM4, which is a part of the LSm2-8 complex. A key difference is that the NSCLC vulnerabilities presented here are from the viewpoint of looking not only at single genes but biological systems such as protein complexes. In contrast with previous analyses, we obtain cell line subgroups that may represent particular patient populations along with candidate targets for each of those subgroups.

## 5 Summary

Novel candidate drug targets were found from computational analysis of a whole genome RNAi knockdown screen in NSCLC cell lines. The targets are protein complexes specific for particular lung cancer cell lines and function in splicing, translation and protein folding. Results of previous studies support further investigation of these protein complexes as avenues of therapeutic intervention in NSCLC. Moreover, the candidate targets provide an opportunity for drug repurposing, which could lead to reduced time in the drug development pipeline. Our results simultaneously establish lung cancer cell line subgroups and potentially novel druggable targets that are specific to each subgroup. This study contributes to a deeper understanding of therapeutically relevant events at the molecular scale in NSCLC.

## Supplementary Material

Supplementary Data
