# Supplementary material for: Computational discovery of pathway-level genetic vulnerabilities in non-small-cell lung cancer
Source: Bioinformatics. 2016 Jan 10;32(9):1373–9. doi: 10.1093/bioinformatics/btw010 (PMC4848405; doi:10.1093/bioinformatics/btw010)
Supplement: Supplementary Data [file supp_32_9_1373__index.html]

Computational discovery of pathway-level genetic vulnerabilities in non-small-cell lung cancer — Computational discovery of pathway-level genetic vulnerabilities in non-small-cell lung cancer — Supplementary Data 

# Computational discovery of pathway-level genetic vulnerabilities in non-small-cell lung cancer

## Supplementary Data

files

- Supplementary Data - zip file
