# Supplementary material for: Computational discovery of pathway-level genetic vulnerabilities in non-small-cell lung cancer
Source: Bioinformatics. 2016 Jan 10;32(9):1373–9. doi: 10.1093/bioinformatics/btw010 (PMC4848405; doi:10.1093/bioinformatics/btw010)
Supplement: Supplementary Data [file supp_btw010_suppl_data.zip › Supplementary Tables and Figures.pdf]

Supplementary Tables

H1155	Large cell neuroendocrine
HCC366	Adenosquamous
H1819	Adenocarcinoma
HCC44	Adenocarcinoma
HCC4017	Large cell carcinoma
H1993	Adenocarcinoma
H460	Large cell carcinoma
H2073	Adenocarcinoma
H2009	Adenocarcinoma
H2122	Adenocarcinoma
H1395	Adenocarcinoma
HCC95	Squamous cell carcinoma
HBEC30	Normal bronchiole epithelial

Table S1. Thirteen cell lines on which our whole genome RNAi screen and computational analyses were conducted.

Bicluster rank	Size (genes × lines)	Lines affected	Enriched annotations
1	1591 x 12	all but HBEC30	Translation, splicing, kinetochores, mitosis
2	756 x 1	HBEC30	No functional enrichment
3	1060 x 1	HCC4017	Translation, splicing, nuclear lumen
4	1141 x 1	HCC366	Nuclear proteins, proteasome non-ATPase subunits
5	1219 x 1	H1819	Translation, splicing
6	813 x 1	H1155	Nucleolar, cytoskeletal proteins
7	1154 x 1	H2073	Wnt pathway
8	859 x 2	H460, H2122	No functional enrichment
9	920 x 1	H1395	Translation
10	1254 x 1	H1993	No functional enrichment
11	1629 x 1	HCC95	Translation, splicing, lysosomal ATPase
12	450 x 2	HCC44, H2009	No functional enrichment
13	104 x 9	9 lines	Ribosome, splicing, nuclear lamin, proteasome
14	159 x 2	H460, H2009	No functional enrichment
15	119 x 2	H2009, H2122	No functional enrichment
16	216 x 2	HCC44, H460	No functional enrichment
17	177 x 2	HCC44, H2122	No functional enrichment
18	66 x 3	H1155, H2073, H1395	No functional enrichment
19	74 x 4	H1155, H2009, H1395, HCC95	Ribosome, proteasome, COPI transport
20	120 x 3	H1993, H2073, H1395	No functional enrichment
21	14 x 5	5 lines	No functional enrichment
22	20 x 7	7 lines	Splicing

Table S2. All statistically significant biclusters (Bonferroni-corrected $P < 10^{-5}$) discovered from Large Average Submatrix (LAS) biclustering. Functional enrichment as discovered through DAVID also shown.

Bicluster rank	Number of genes in bicluster	Number of enriched protein complexes	Significant enrichments (p-value)
1	1591	71	Spliceosome (2.1×10^{-43}); 40S ribosomal subunit (2.3×10^{-16})
2	756	4	20S proteasome (5.5×10^{-5})
3	1060	30	Spliceosome (1.5×10^{-15}); Nop56p-associated pre-rRNA complex (5.4×10^{-11}); NRD complex (8.0×10^{-6})
4	1141	21	NuA4/Tip60-HAT complex B (2.1×10^{-5}); PA700-20S-PA28 complex (1.7×10^{-6})
5	1219	24	Spliceosome (2.9×10^{-13})
6	813	2	109-member metabolic and motor protein complex (1.1×10^{-4})
7	1154	2	PID complex (1.4×10^{-4})
8	859	10	Spliceosome (1.5×10^{-11})
9	920	5	140-member ribosomal protein complex (1.8×10^{-9})
10	1254	0	No enrichment
11	1629	25	Spliceosome (1.8×10^{-17})
12	450	1	SAR1A-TPD52 complex (8.9×10^{-5})
13	104	50	Spliceosome (8.6×10^{-13})
14	159	2	Nop56p-associated pre-rRNA complex (5.2×10^{-7})
15	119	0	No enrichment
16	216	18	Spliceosome (4.4×10^{-6})
17	177	0	No enrichment
18	66	0	No enrichment
19	74	23	PA700-20S-PA28 complex (2.8×10^{-18}); 40S ribosomal subunit (5.1×10^{-7})
20	120	0	No enrichment
21	14	0	No enrichment
22	20	3	C complex spliceosome (4.2×10^{-8})

Table S3. Protein complex enrichment of bicluster genes discovered from LAS biclustering. Enrichment was computed from the hypergeometric probability of obtaining at least the observed amount of overlap between protein complex and bicluster genes. All enriched protein complexes are statistically significant at 5% FDR as determined by the Benjamini-Hochberg procedure. For brevity, statistically significant complexes with biological functions also found to be enriched with DAVID are shown.

Supplementary Figures

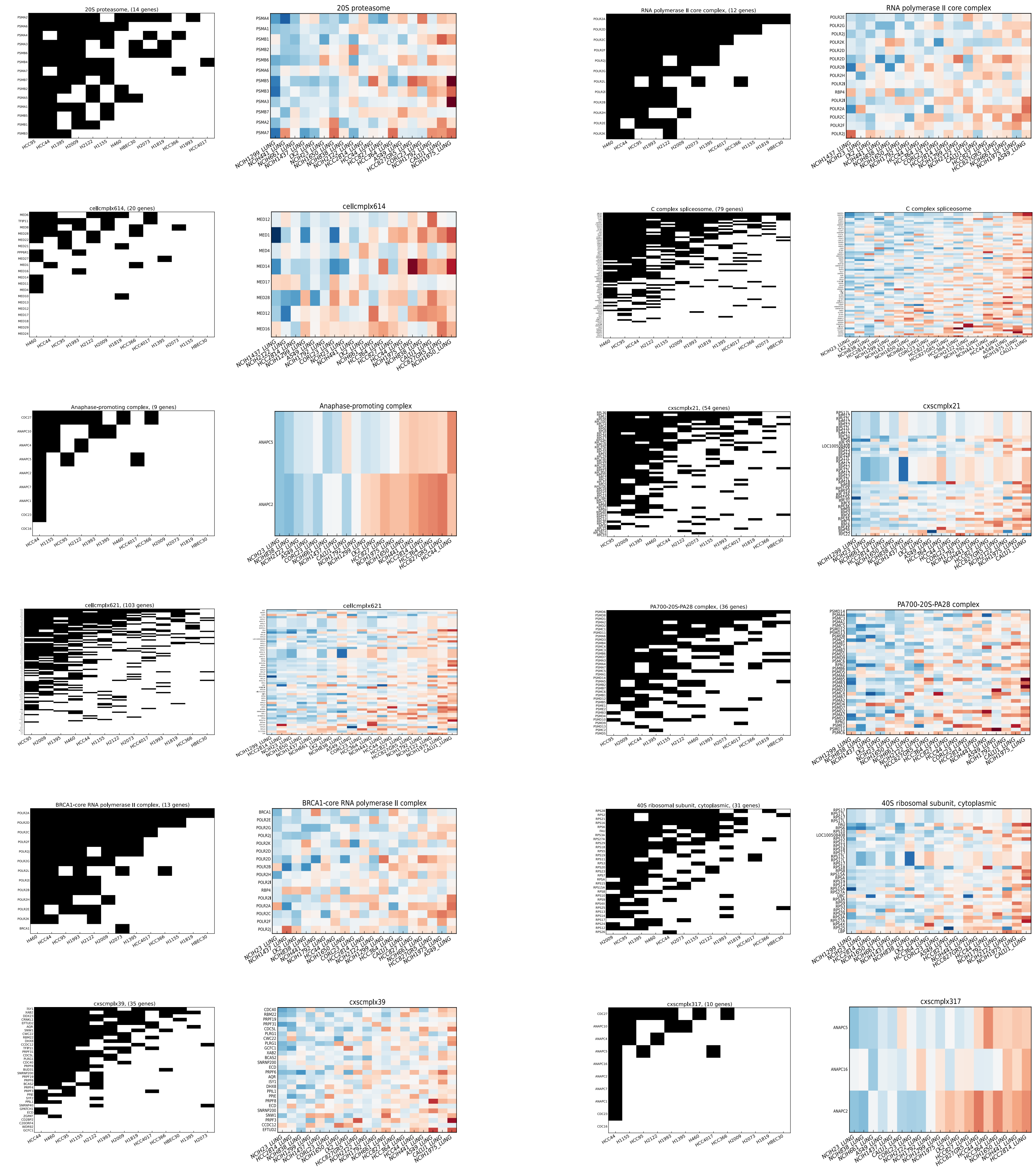

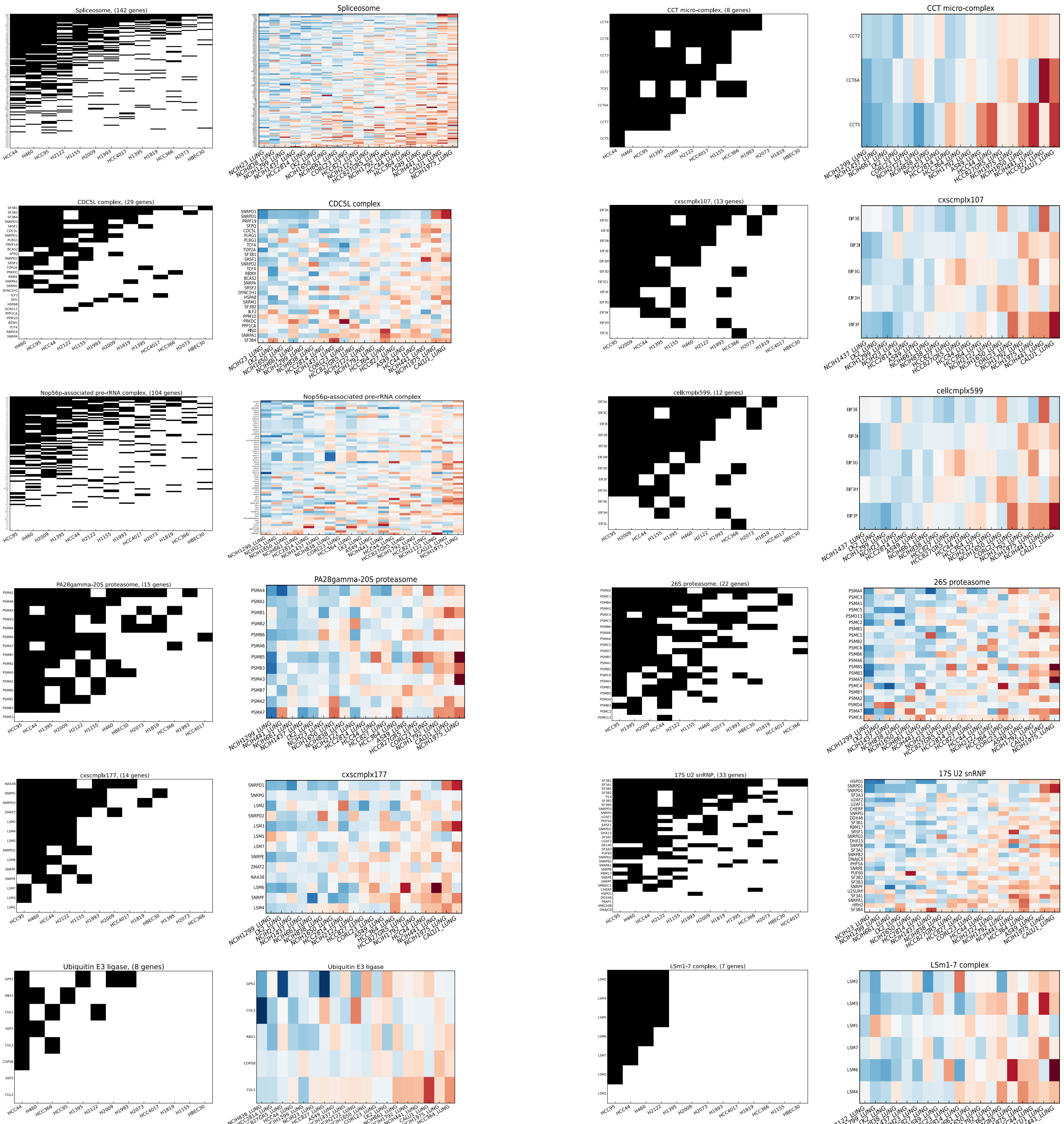

Spliceosome, (142 genes)
Spliceosome
CCT micro-complex, (8 genes)
CCT micro-complex
CDC5L complex, (29 genes)
CDC5L complex
cxscmplx107, (13 genes)
cxscmplx107
Nop56p-associated pre-rRNA complex, (104 genes)
Nop56p-associated pre-rRNA complex
cellcmplx599, (12 genes)
cellcmplx599
PA28gamma-20S proteasome, (15 genes)
PA28gamma-20S proteasome
26S proteasome, (22 genes)
26S proteasome
cxscmplx177, (14 genes)
cxscmplx177
17S U2 snRNP, (33 genes)
17S U2 snRNP
Ubiquitin E3 ligase, (8 genes)
Ubiquitin E3 ligase
LSm1-7 complex, (7 genes)
LSm1-7 complex

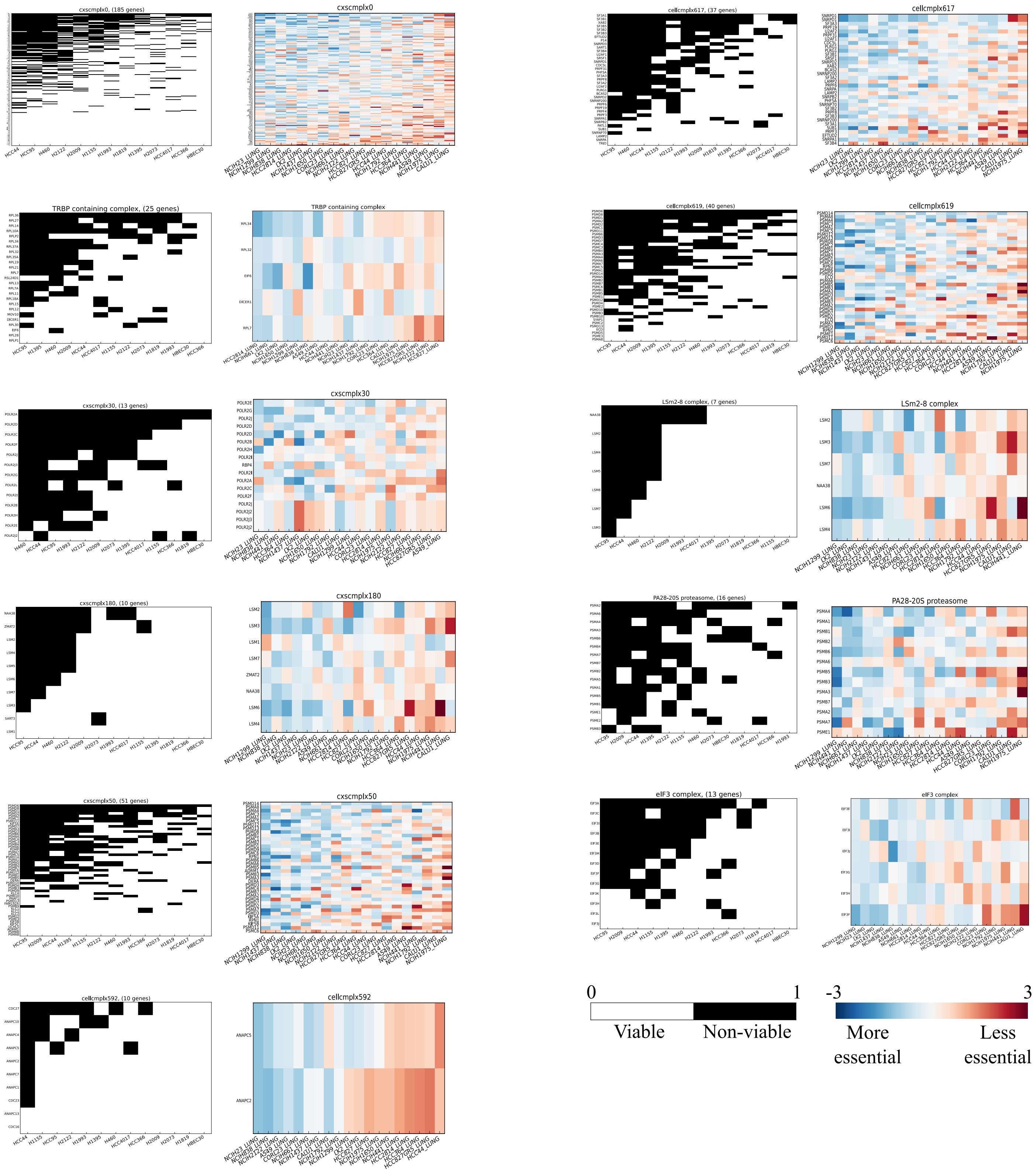

Figure S1. Genetic vulnerability patterns of protein complexes.
Shown are all 35 protein complexes found from the 2-means clustering approach to be statistically significant at 10% FDR. Also shown for each complex is its corresponding shRNA knockdown sensitivity profile from Project Achilles. Note that some genes in certain protein complexes are absent in the Project Achilles heatmaps, which had knockdown data available for only 5711 genes. There are also some genes with multiple knockdown values for each cell line in Project Achilles. Complex labels indicate source (cellcmplx, Havugimana *et al.*, 2012; cxscmplx, http://metazoa.med.utoronto.ca).

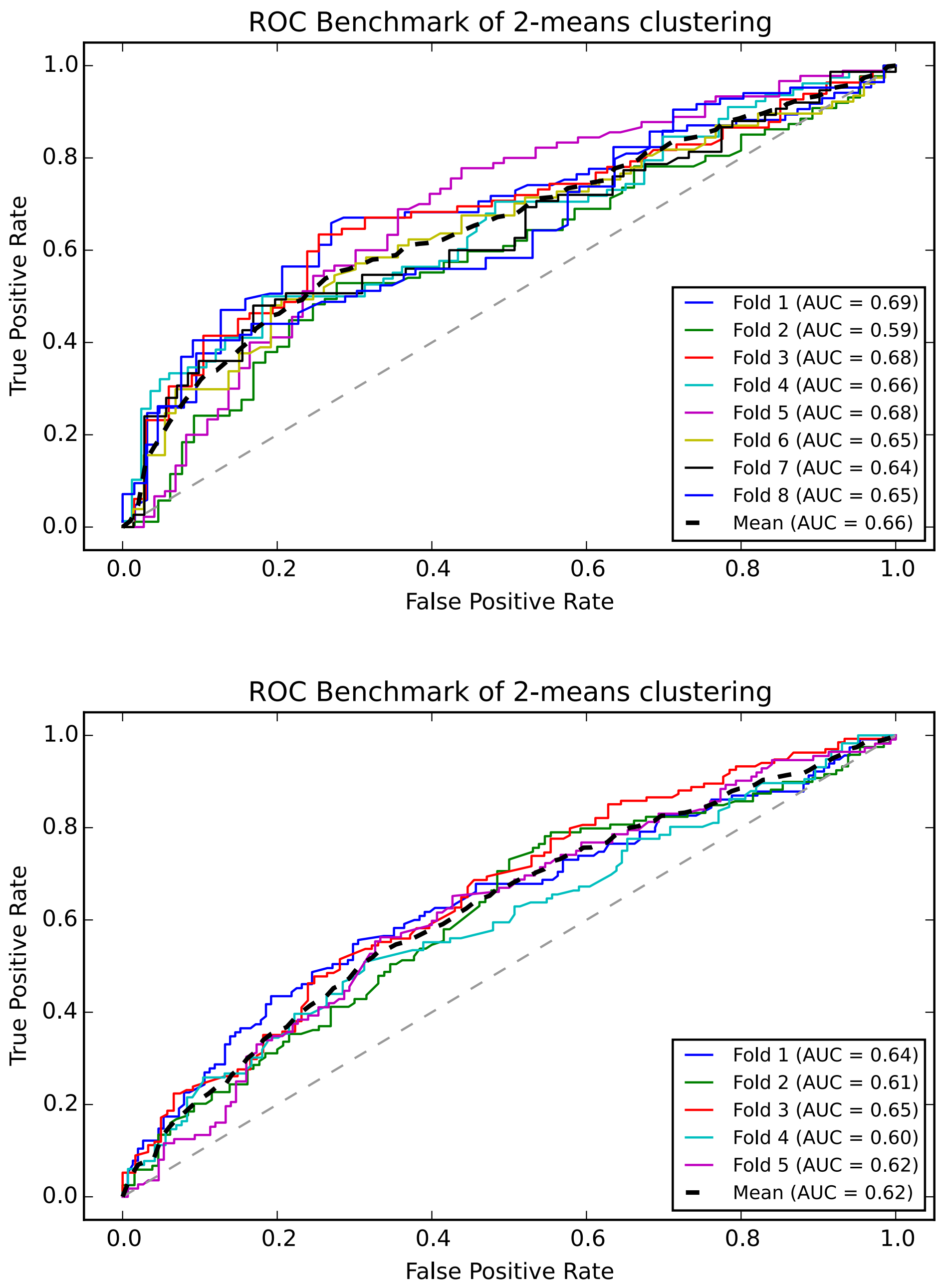

Figure S2. Receiver operating characteristic (ROC) benchmarking of 2-means clustering. The top plot was obtained from 8 iterations of a leave-one-out benchmarking scheme for protein complexes with at least 8 members. Each iteration, a gene member was randomly withheld from each complex. 2-means clustering of the remaining members predicted average sensitivity vs. resistance of cell lines in the withheld test gene. The bottom plot shows the analogous results for 5 iterations when restricting to complexes with at least 5 members.

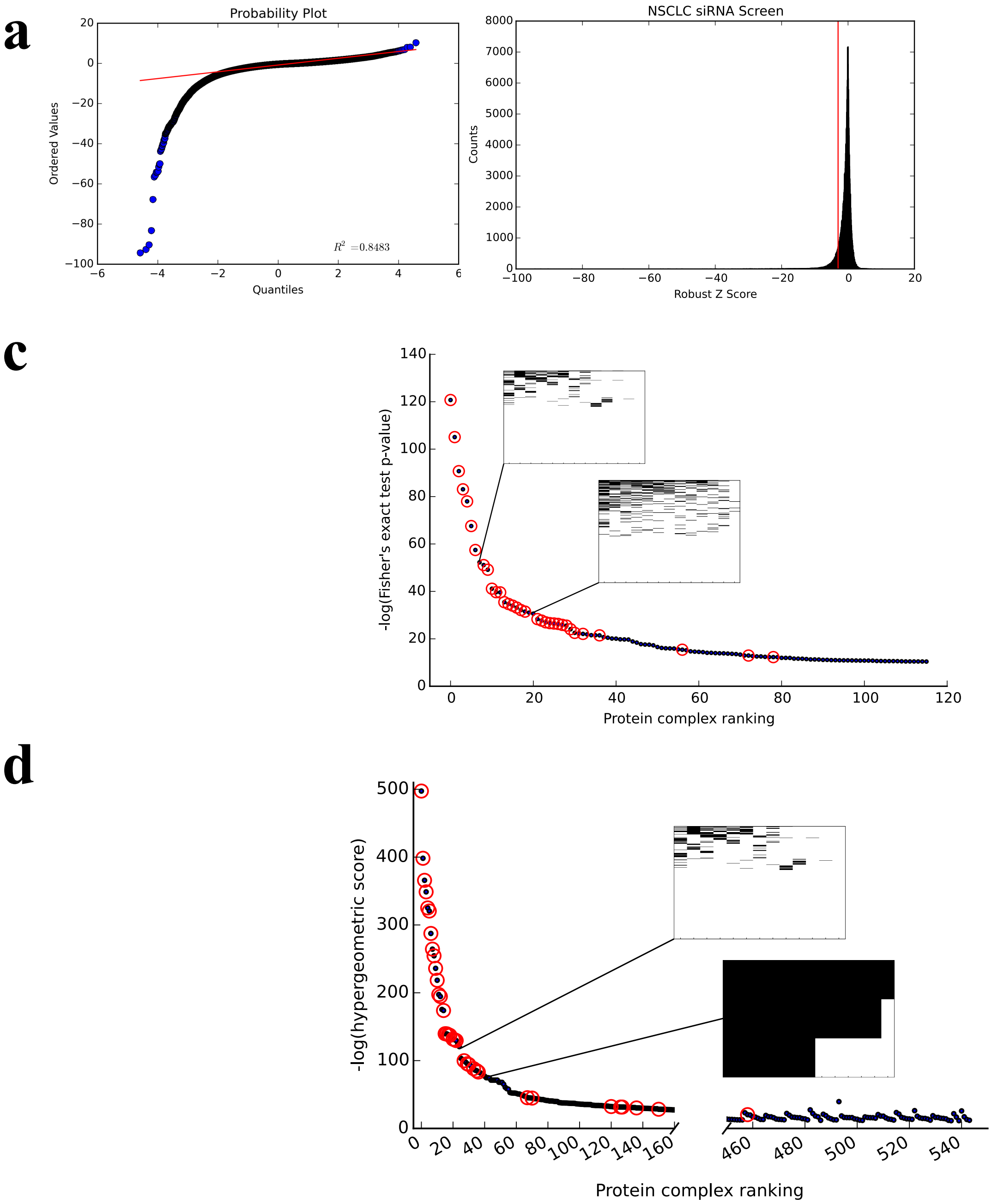

Figure S3. Alternative approaches to measuring complex sensitivity.
(a) Q-Q plot (left) shows that the robust Z scores from the whole genome siRNA screen are not normally distributed. The score distribution (right) is skewed; scores to the left of the red line indicate non-viability.
(b) Even if the data were normally distributed, a z-test comparing complex sensitivity to the background distribution would not distinguish a half-toxic complex (left) from a bimodal complex (right).
(c) Circled in red are 33 of the 35 protein complexes from the permutation test to indicate where they rank according to Fisher's exact test, which discovered 116 significant complexes at 10% FDR. Also shown are two complexes not prioritized by the permutation test.
(d) Circled in red are the 35 protein complexes from 2-means clustering to indicate where they rank according to an alternative hypergeometric scoring method, which discovered 544 significant complexes. Both methods used a permutation test and *q*-value to control the FDR at 10%. Also shown are two complexes not prioritized by 2-means clustering.

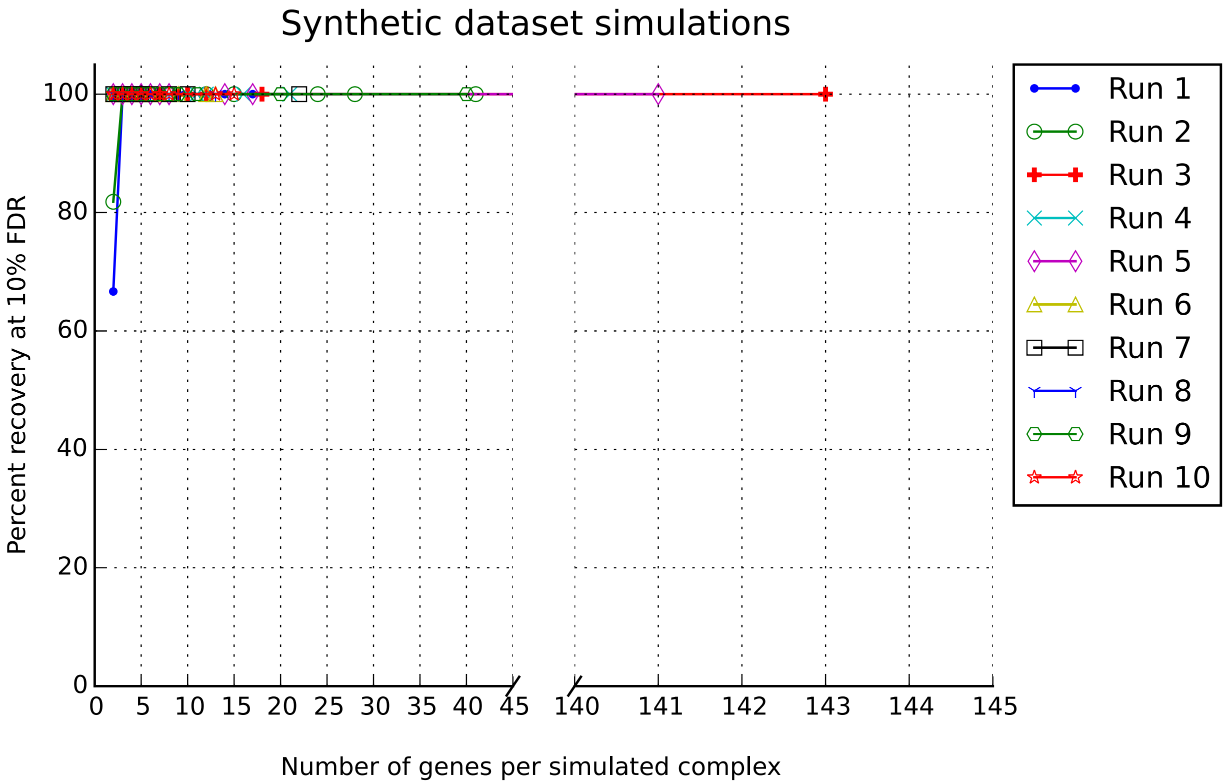

Figure S4. Simulations of 2-means clustering and permutation testing on a synthetic dataset. To evaluate whether permutation testing for statistical significance of 2-means clustering scores is biased towards larger or smaller complexes, simulations on synthetically generated data were conducted. A 90% random sparse matrix of ones and zeros with the same dimensions as the actual RNAi data (24866 rows and 13 columns) was constructed. Each simulation run consisted of randomly chossing 200 submatrices with the row size of each submatrix drawn from the same size distribution as that of the actual protein complexes. Therefore, each submatrix simulates a protein complex, with the number of genes in the simulated protein complex corresponding to the number of rows in the submatrix. Of those 200 submatrices, 30 were constructed to be bimodal by setting between 4 and 7 columns equal to 1. The 2-means clustering and permutation test were run on all 200 submatrices. The entire procedure just described constitutes one simulation run. For every simulation run, the percentage of bimodal submatrices that were called significant at 10% FDR was calculated for the various complex sizes. Shown is a plot over 10 simulation runs; the permutation scheme approach is not biased toward larger or smaller complexes, particularly for complexes with more than 2 genes.
